# Supplementary material for: A Qualitative Exploration of Challenges and Opportunities for Dog Welfare in Ireland Post COVID-19, as Perceived by Dog Welfare Organisations
Source: Animals (Basel). 2022 Nov 25;12(23):3289. doi: 10.3390/ani12233289 (PMC9739681; doi:10.3390/ani12233289)
Supplement: Supplementary file 1 [file animals-12-03289-s001.zip › animals-1999471-supplementary.pdf]

Dog welfare organisation Online Interview

Recording Starts:

Thank you for taking the time to meet us today, we really appreciate it. Everything you say is anonymised. You have received a participant information sheet which contains information about your consent to participate. In a short summary, this interview will be recorded, and the information transcribed. This data will be confidential; unless there are serious breaches of good practice, criminal revelations or disclosures of risks of harm to yourself or others. You are not identifiable from any of the information, which may be published in the future. Can you each confirm: your name, that you have understood the participant information sheet and consent to all the statements from that please.

(Individuals will be asked to leave the group if there is no consent to record the interview)

So, we will be asking around 2 main topics today; general dog and dog welfare in Ireland, and the role of dog welfare organisations in Ireland. This project is really focused on helping both and your expertise will be invaluable.

Icebreakers

1. So, to start us off can you tell me a little about the dog welfare organisation you are a part of.
    a. *Prompts:* Where, how many dogs, staffing (qualifications)?

Challenges: Dog Welfare

2. So today, we are looking to identify some of the main challenges for dog welfare, so the overall dog population in Ireland. Can you identify some of the main challenges for this over the next year/18 months?
    a. *Interviewer note:* Get 3 challenges
3. Ok, so in the more medium/long term (3-5 years), what do you see as the challenges in this time period for dog welfare (dogs in general)

Challenges: Dog Welfare Organisations

4. Ok, so for organisations such as yours, what challenges do you see over the next year/18 months?
    a. *Prompts:* Get 3 challenges
5.
6. In the more medium/long term (3-5 years) for organisations such as yours?
    a. *Interviewer note:* Get different ones than short terms.

Solutions to challenges

7. As an organisation, what steps do you think would help:
    a. Dog welfare in general
    b. you as an organisation?
        a. *Interviewer note: Focus on local solutions, then national.*
        b. *Interviewer note: Move it beyond the more money idea.*
8. Finally, on this part, has anything over the past 5 years helped significantly for dog welfare

a. *Prompts:* Either policy or you have done.

<u>Using grants and money</u> (not for Dogs trust)

9. As a funded/unfunded organization, how do you use the grants to fulfill your mission?
    1. Do you feel that the money is being spent appropriately?
        a. *Prompts:* Appropriate Nutrition
        b. *Prompts:* Equipment
        c. *Prompts:* Especially vet fees and QALYs (no-hopers).
        d. *Prompts:* Normal donation fees and use
        e. *Prompts*: Staff or owner education?
10. Do you use funding on more strategic or longer-term measures?
    1. Such as prevention or proactive measures?

<u>Best practice</u>

11. As part of this project, we are looking to develop best practice guidelines for dog welfare organisations. Can you tell me what you think the main items should be on it?

    a. Prompt: Get both care and governance/management perspectives.

    b. entry of dogs, residence, exit [i.e. rehoming]), but also broader governance and management (finances, oversight, strategic planning etc.).

    c.

<u>Hot button issues</u>

12. One thing raised recently in the media, has been the exporting of dogs to other countries (such as Britain and Sweden) by dog welfare organisations?
    a. Does your organisation export dogs?
    Interviewer note: Remind respondents of confidentiality.
    b. Why do you think this occurs?
    d. Do you think there is a financial element at play?
    e. Are there any breeds in particular that are being exported?
    e. Why do you think this happens?
13. Covid brought up challenges and opportunities. Can you tell me some more about these <u>for?</u>
    a. Has demand changed
    b. Breed as well
    c. Post-COVID issues
14. Have you heard of the *W*orking *Together for Animal Welfare Ireland's Animal Welfare Strategy 2021-2025'?*
    a. Within this, what priorities are most closely aligned to your mission/organisation?
    b. Has the strategy made any changes to your practice since its inception/start?

Thank you very much for your time today. Your expertise and experience will be a great benefit in the development of better welfare standards and helping dogs in Ireland.
